# Midwives experiences of meeting pregnant women who are exposed to Intimate-Partner Violence at in-hospital prenatal ward: A qualitative study

**DOI:** 10.18332/ejm/125941

**Published:** 2020-09-15

**Authors:** Hafrún Finnbogadóttir, Ella Torkelsson, Cecilia B Christensen, Eva-Kristina Persson

**Affiliations:** 1 Department of Care Science, Faculty of Health and Society, Malmö University, Malmö, Sweden; 2 Department of Obstetrics and Gynaecology, Malmö University Hospital, Malmö, Sweden; 3 Department of Health Sciences, Lund University, Lund, Sweden

**Keywords:** pregnancy, content analysis, intimate-partner violence, antenatal care, focus-group interviews, in-hospital prenatal ward

## Abstract

**INTRODUCTION:**

Worldwide every third women is exposed to physical and/or sexual violence and pregnancy is no safe period for the women. The aim was to elucidate midwives experience of violence-exposed pregnant women who had been referred to a prenatal ward and were hospitalized.

**METHODS:**

An inductive qualitative method was used with four focus-group interviews performed with sixteen midwives working at in-hospital prenatal ward. The data were analyzed with content analysis.

**RESULTS:**

Three categories emerged. ‘Professional area of responsibility’, the midwives working at in-hospital prenatal ward considered it was the responsibility of the midwives working at antenatal care to ask routinely in order to detect violence-exposed women. Signs of help-seeking were based on the pregnant woman’s behavior. Suspicion of intimate-partner violence was based on gut feeling. ‘Conditions for support’, the midwives strived to support pregnant women who were already identified as violence-exposed or if they had a suspicion that the pregnant woman was in a relationship where intimatepartner violence occurred. ‘Barriers for giving support’, both the work-place layout and routines constituted a barrier. The midwives own emotional state could affect her handling of the situation.

**CONCLUSIONS:**

The midwives working in-hospital considered it the responsibility of the midwives at antenatal healthcare to identify these women. The midwives had limited experience in dealing with violence-exposed pregnant women but recognized a number of signs and symptoms that could cause suspicion. They felt uncomfortable in the situation and expressed a need for both education and an action plan.

## INTRODUCTION

Worldwide every third woman is exposed to physical and/ or sexual violence^1^. About 20% of women in Sweden have reported being exposed to psychological, physical or sexual violence by a current intimate-partner or an earlier intimatepartner^2^. According to World Health Organisation (WHO), violence against women is not only a public health problem, but also violation against human rights. Pregnancy makes women more vulnerable for intimate-partner violence (IPV) than ever, due to changes in the physical, emotional, social and economic situation^1^. A report from WHO^3^ showed that globally at least 5% of pregnant women have been exposed to physical violence by their intimate partner. The perpetrator was in 90% of the cases the biological father of the unborn baby. Prevalence of domestic violence (DV) against women during pregnancy in high-income countries is estimated to be 13.3%^4^. In Sweden, the prevalence of violence against pregnant women is reported to be between 1–11%^5-7^. To find the women who are exposed to DV during pregnancy, midwives at in-hospital prenatal wards have an important role to play.

The literature diverges regarding whether the violence is unchanged during pregnancy^8,9^, starts^3,10^, increases^11,12^ or decreases, albeit not uniform across all cultures^3^. Brownridge et al.^13^ reported that pregnancy may be a protective factor, but those women who already were exposed to violence during their pregnancy had greater risk of being exposed to all types of violence and especially rougher violence. A study from Sweden reported that the prevalence of DV increased from 1% in early second trimester to 2.5% at the time-point for birth^5^. Finnbogadóttir et al.^14^ showed that violence-exposed women are reluctant to leave the perpetrator during the pregnancy and struggle to survive for the sake of the unborn baby. Even though, the thought of the unborn baby may give the mother-to-be a hope for a change in her life^14,15^. Due to shame and guilt, pregnant women chose not to reveal their difficult situation to anyone^14,15^. Spangaro et al.^16^ showed that a direct clear question from a midwife, who the women trusted, could be a turning point to talk about the violence. This is in line with the description of a good midwife by Halldórsdóttir and Karlsdóttir^17^ who among other things, described a good midwife’s interpersonal competence as capable of empowering communication and partnership with the woman and her family.

A history of violence, to be single, or living apart^4,11^, having a low education level, and an unplanned pregnancy, have been identified as risk factors for exposure to DV during pregnancy^4^. A systematic review demonstrated the association between prenatal depression, anxiety, post-traumatic stress disorders and a history of violence, especially during pregnancy^18^. Also, a bigger longitudinal cohort study from Sweden supports a strong association between being exposed to violence during pregnancy and depressive symptoms^11,19^.

Personal exposure to violence during pregnancy has a negative impact on both the mother-to-be and the unborn baby’s health^14,18^. An increased risk of hospitalisation due to obstetrical complications^20,21^ and caesarean section^21,22^ has been shown. There is an increased threat of abortion before gestation week 22, premature rupture of membranes, antepartum and postpartum bleeding^23^, as well as an increased risk for premature birth, small for gestational age^24,25^ and low-birthweight babies^25^. An association has also been reported between exposure to violence during pregnancy and depressive symptoms^5,18,20^.

Taft et al.^26^ made a systematic review of the literature that supports that a routine enquiry about DV during pregnancy increases the rate of detection, but there is insufficient evidence for the long-term benefits of asking routinely^26^. Another systematic review by Jahanfar et al.^27^ reported insufficient evidence regarding the effectiveness of interventions for DV in relation to pregnancy outcomes.

In Sweden, midwives have an important role in both identifying IPV and preventing all sorts of health risks^28^. In order to increase the prerequisite of detecting IPV, it is recommended that all women attending antenatal care should be asked routinely about their experiences of violence. A plan of action when the women answer positively should exist^29^. The Swedish Society of Obstetrics and Gynecology (SFOG) (2016) also stipulates that all pregnant women should be offered at least one individual visit without her partner, during the antenatal care period.

It is difficult to identify a pregnant woman exposed to violence by meeting her only once^30^. Lack of personnel and time, uncertainty about how to ask the relevant question^31^ and the partner’s presence^32^ are barriers to identifying vulnerable women who are exposed to IPV. Finnbogadóttir et al.^32^ also showed that the midwives own experiences and attitudes could be obstacles. They also pointed out that the midwife can be worried about how a trusting relationship with the pregnant woman may be affected. The lack of education, written guidelines and how to document cases resulted in difficulties for the staff to cope in situations where violence was disclosed. However, midwives consider the question about the exposure of IPV an important task and that they feel they will let the woman down if they do not ask the sensitive questions (ibid). A systematic review by O’Doherty et al.^33^ showed that asking routinely increases identification of women who are vulnerable and pregnant, and they are more likely to reveal their situation to a midwife than in any other healthcare situation. Utilization of healthcare services by violence-exposed women is high and may provide the only contact opportunity with authorities^1^. The healthcare system has a significant responsibility to reveal exposure to violence and offer support and treatment^34^. According to the National Board of Health and Welfare regulations and general advice about IPV, all healthcare personnel have to ask questions about exposure to violence when suspicion arises^28^. The aim of the present study was to elucidate midwives experience of violence-exposed pregnant women who are referred to an in-hospital prenatal ward.

## METHODS

An inductive qualitative method was chosen. The data collection was performed with focus-group interviews^35^. The inclusion criteria were midwives who had clinical experience at an in-hospital prenatal ward for at least one year. By using focus-group interviews, the midwives were encouraged to discuss with each other, as freely as possible, their clinical experience of pregnant women who were patients at inhospital prenatal wards and possible survivors of IPV. The interviews were analysed with thematic content analysis according to the Burnard et al.^36^ study.

### Setting and participants

Midwives from four in-hospital prenatal wards from different geographical areas in southern Sweden were recruited. One of the wards was specialized in prenatal obstetrical complications and observations, while the others were combined prenatal and postnatal care.

### Recruitment

The information about the study was given both orally and written at a workplace meeting by the two interviewers. No recruitment took place at that time. The heads of the inhospital prenatal wards acted as gatekeepers and suggested a date and time for the focus-group interview, which fitted the work load. Thereafter, the expected participants were contacted by the interviewers to ask if they wished to participate in the study. In direct connection to the focusgroup interview, the participants gave their written consent. The interviews were conducted at the workplace and during working hours. The Advisory Committee for Research Ethics in Health Education at Lund University, Sweden, provided an advisory statement prior to the study (Diary number: VEN 41-16). The study was conducted in compliance with the ethical principles of the Declaration of Helsinki^37^. The respondents were informed that participation was voluntary and that they could withdraw their participation whenever they wished.

### Data collection

Four focus groups with 2–7 midwives in each were conducted and totally sixteen midwives participated.

The respondents had work experience of 3.5–34 years (mean 13 years) and had worked in perinatal wards for 2–25 years (mean 11 years). The majority had clinical experience in caring for violence-exposed women. Three of the respondents had only theoretical knowledge and no personal experience of meeting violence-exposed women. All the interviews were performed by two of the authors (ET & CBC) and they alternated as a moderator and an observer. The interviews started with the overreaching question: ‘Would you please tell us about how you, as a midwife, work with pregnant women who are exposed to intimate-partner violence?’. Follow-up probing questions were used, such as: ‘Could you please tell me more about that situation?’ or ‘Can you please develop this further?’. The interviews were audio recorded and lasted between 30 to 52 minutes with a median of 44 minutes.

### Data analysis

The data analysis was performed using content analysis inspired by Burnard et al.^36^. Initially, each interview was listened to several times to get a perception of the content. Thereafter, the interviews were transcribed verbatim and were read through thoroughly several times. Notes about the character of the text were created in the margin. Superfluous text was considered as marginal.

An initial coding framework from the interview transcripts was made to facilitate further data processing. Open-coding, theories or phrases that summed up what was being said in the text was performed. Two of the authors (ET & CBC) completed the initial open-coding with support from the first author (HF). The final coding framework was made after reduction of the categories in the initial coding framework. Afterwards, all the co-authors discussed the coding and analysis of the text material, dividing it into subcategories, categories and themes, until consensus was reached. Thereafter, the coherent text, which describes the content of the categories, was written together. Finally, quotations that captured the essence, from both the individual respondents and the dialogue interactions, were chosen from the entire text to confirm credibility.

## RESULTS

Three categories, with three subcategories each, emerged in the analyses. The categories were: ‘Professional area of responsibility’, ‘Conditions for support’ and ‘Barriers for giving support’ (Table 1).

**Table 1:** Categories and subcategories of responsibility and support

*Professional area of responsibility*	*Conditions for support*	*Barriers for giving support*
Displacement of responsibility	Generating confidence	Organisational conditions
A cry for help	Offering help	Lacking guidelines and education
Having a gut feeling	Protecting both mother and child	Influenced/affected by own feelings

### Professional area of responsibility

This category included three subcategories: ‘Displacement of responsibility’, ‘A cry for help’ and ‘Having a gut feeling’.

#### Displacement of responsibility

The midwives expressed a sense of security that their colleagues in ANC (antenatal care) are required to ask all women about their experience of DV and meant that it was their responsibility to ask this sensitive question. If documentation from ANC about IPV had been done, their own suspicions could be confirmed. Questions about IPV were not asked routinely at the in-hospital perinatal wards, only when suspicion arose.

‘*I don’t think I have ever asked. We don´t ask - I think it´s done at the ANC where those questions are asked. So you perhaps ask there, but…’*. (Midwife 14)

*‘They should always ask. They ask: Do you smoke? Do you drink alcohol? Have you been exposed to violence? It´s kind of a routine’*. (Midwife 15, Focus group 4)

At the same time, the midwives expressed awareness about the possibility that there was a great number of unreported cases of violence-exposed women. The midwives mentioned that despite official recommendations to ask all women this delicate question, many colleagues still refrained from doing so and that violence-exposed women did not necessarily answer honestly about ongoing abuse. The midwives thought that it may be easier to disclose violence exposure when it had stopped. They also discussed that violence-exposed women most likely had passed through the healthcare system without telling anyone about it.

*‘That you don´t notice it, it´s not because you can´t. Nothing has been done for ages, you think it should have been noticed previously’*. (Midwife 3, Focus group 1)

#### A cry for help

Suspicion could be aroused about IPV if the midwife noticed in the case notes that the pregnant woman continually had failed to arrive to planned antenatal visits or had sought care several times without apparent medical reasons, or if she wanted to be discharged from hospital before it was recommended. The midwives discussed that some women may look for care at several different healthcare clinics. This can be due, on the one hand, to the women’s wish to be anonymous and, on the other, for her wish for the situation to become apparent. If the woman wished confidentiality, could doubts about IPV arise? In the discussions, the midwives kept apart the reason for the original contact with healthcare and the eventuality of exposure to IPV. It was expressed that inpatient-care pregnant women were admitted due to pregnancyrelated complications. At the same time, several common pregnancy-related complications were mentioned as possible effects of IPV, such as reduced foetal movements, bleeding or hyperemesis. Visible injuries such as bruises or burns on the body, face or arms appeared as clear signs of violence in a close relationship. When the woman was asked about the injuries, she often glossed over them by saying that she had fallen. The midwives identified pregnant women with substance abuse, those with psychological ill health or foreign born, as special exposed groups.

*‘… they have really vomited up to the end of pregnancy and it was just anxiety and fear that made them vomit so much and they couldn´t differentiate between hyperemesis or if it was something else. Something else behind it’*. (Midwife 7)

*‘They get a chance to be away from home, be in a safer situation because they are often in hospital for a long time’*. (Midwife 11, Focus group 3)

#### Having a gut feeling

A suspicion about violence in close relationships was often based on a feeling that arose from the behaviour of the woman, partner, or the couple. The midwives considered themselves to have an inherent ability to interpret the woman’s and her partner’s body language, tone of voice, emotional state, and the interaction between the couple. The feeling was based on intuition, knowledge about violence in close relationships, and previous experience. Concurrently, the midwives were aware that the feelings were subjective and that there was a risk of misinterpretation.

*‘…you get a feeling, you know, I think, that work with this are slightly more receptive for emotional feelings. Just feelings. It´s so we can ´read´ the patient, ´read’ the relatives. You kind of sense their frame of mind. You kind of feel in the atmosphere that something is not quite right’*. (Midwife 8, Focus group 3)

During the discussions, it was expressed that violence may often be subtle. When the pregnant woman was silent, acted in a restrained or repressed way, a suspicion could arise. The pregnant woman could be reserved or having difficulties in being or coming alone. The midwives thought they saw a connection between a difficulty or dislike at being touched and a history of violence in a close relationship. A perpetrator was described as manipulative and someone who tried to control the pregnant woman. Often the partner behaved dominantly and frequently brought forward the woman’s case concerning the care. Sometimes the partner was perceived as being directly threatening to both the woman and the staff.

*‘Always there, they can never be alone and things like that. She can maybe say something that is negative for him or her. I think it happens often, very, very often… The becoming father simply talks over her head. It is he that comes and says how we have done this, and she has done that. It´s never anything she says, it’s always he that speaks’*. (Midwife 9, Focus group 3)

Evaluation of the couple’s relationship became more challenging for the midwife if there was a language barrier. The midwives expressed how important it was to use a certified interpreter when talking to a woman who had not mastered the Swedish language, so as to ensure that the partner did not influence the woman’s answers.

### Conditions for support

This category has three subcategories: ‘Generating confidence’, ‘Offering help’ and ‘Protecting both mother and child’.

#### Generating confidence

It was important to create a trusting relationship with the pregnant woman if the midwife suspected that the intimate partner exposed her to violence. The midwives could approach the woman by being present in her room or being more personal by talking about everyday things. Furthermore, confidentiality could be achieved by highlighting that the personnel are bound by confidentiality for their patients. When a trusting relationship had been created, the midwife could ask the pregnant woman about possible IPV.

*‘… then you try to sit with the patient, to try and develop a relationship so you can get round to asking the question and you will get an honest answer. You need an honest answer and for that you need some kind of relationship’*. (Midwife 13)

*‘If you don´t have a good relationship it´s not possible’*. (Midwife 10, Focus group 3)

The questions could be expressed in different ways, through a statement, a straight question or by carefully approaching the subject. It was considered important to ask the question when the partner was not present. Sometimes it could be difficult to find an opportunity to talk to the woman alone because the partner was encouraged to be present as much as possible. The midwives expressed concern that the partner would become aware of their suspicions and that the woman’s exposure to violence could increase.

*‘But I think it´s often very difficult, because you want some kind of contact if you get this feeling and experience. Ah. You want to connect with her to sit down and talk to her.*

*But it´s very difficult to get contact with her just because we want information. If we do, she maybe suspects and then we have caused a bigger problem. More. That she is exposed to more violence. Why shouldn´t he be here?’*. (Midwife 16, Focus group 4)

#### Offering help

The midwives considered that it was their duty to inform the pregnant woman about possible areas for help in the event of established or suspected violence. They could, for example, refer her to a welfare officer, women’s aid or social service worker, and, with the women’s permission, help her to contact the police. When the midwives had a strong suspicion that the woman had been exposed to violence, it was frustrating when the woman declined help, because the midwives were not able to act against the woman’s wishes. Also, in case of established violence in a close relationship, it occurred that the woman refused to be offered help. Although the midwives expressed a sense of insufficiency, they usually felt they could help the hospitalized woman. They saw it as their task to support and strengthen the pregnant woman during the care period. By highlighting the woman’s value and ability, they felt they could ‘saw a seed’ wish that in turn could lead to a possible change in the pregnant woman’s life.

*‘We are midwives, we deliver, and we have our ways to try and solve the situation. That’s how we are, to always act. To help, help, help etc’*. (Midwife 4)

*‘Or how? It’s like that for people early in their lives and there are a lot of horrible things that happen if you don´t do something so I think you have an inbuilt protective instinct. It shouldn´t be like this… It´s something that is very much against the norm and something has to be done even if the woman doesn´t want help I think that it always says this isn´t normal… You can say you open a door, you open the door and say “there´s a high ceiling” here we can talk about it’*. (Midwife 2, Focus group 1)

#### Protecting both mother and baby

The midwives had a strong desire to protect both the woman and the unborn baby. They considered themselves to have greater authority to act for the unborn baby than for the woman. When there was already another child in the family there was an obligation to report to the social services, which the midwives felt supported their actions. It occurred that notification to the social services was performed during the pregnancy and the midwife found it frustrating that any action first took place when the baby was born. In addition, the woman could perceive a report to the social services as a threat, because she thought it could lead to her not being able to keep the baby after birth. The midwives meant that it was difficult to make a report based on a feeling or suspicion. They felt that acting for the best of the baby did not imply a better situation for the mother. They also felt anxiety that the violence against the woman could increase if the partner became aware that a report had been made.

*‘No, but it feels as if we have more authority as far as children are concerned. It´s like this, even if the parents don´t think it´s alright so can we say “No, we will make a report, we want the social services to come”. But you don´t do this if the woman is exposed to violence from her man’*. (Midwife 14, Focus group 4)

### Barriers for giving support

This category has three subcategories: ‘Organisational conditions’, ‘Lacking guidelines and education’ and ‘Influenced/affected by own feelings’.

#### Organisational conditions

The midwives felt that the conditions at the workplace could be an obstacle to give care to a suspected or already identified violence-exposed pregnant woman. It appeared that midwives had insufficient time at work for all commitments and subsequently also a lack of time to observe for possible signs of violence. High workload meant that medical procedures were prioritized. The midwives managed to give this delicate task attention only by re-prioritizing their work and extending the care period as well as receiving help from colleagues. During medical rounds and when reporting to colleagues, the midwife was able to convey her suspicions to other midwives, doctors and nurses. The design of the wards enabled free access for visitors and could lead to an uncertain situation for both the pregnant woman and the midwife in the event of a direct threat. One obstacle to being able to act was perceived to be that today’s care is highly specialized and that there was the risk of the woman ‘falling between the chairs’.

*‘Yes, you don´t have much time to sit down and talk. We don´t have time’*. (Midwife 5)

*‘It´s such a little thing. When you see something, you have to deal with you must know you have the time it takes’. (Midwife 6). ‘Yes’. (Midwife 5). ‘And there´s not always that so maybe we close our eyes instead. I think it´s very possible this happens’*. (Midwife 6, Focus group 2).

#### Lacking guidelines and education

The midwives stated that they lacked knowledge of violence in close relationships. The majority had not received training about the subject during their midwifery education. The employer offered no further training on the subject, so the midwives themselves had to take the initiative to gain more knowledge. They experienced a lack of up-todate guidelines and could feel perplexed when they had a suspicion or identified a pregnant woman exposed to violence. No matter what knowledge they had, the midwives felt a responsibility to act. The midwives had a desire to get feedback on their actions in order to be able to confirm that they had acted correctly.

*‘You must have an official plan, that if you start asking this question you must have a plan for what I should do. Because before you have that you can´t start to ask’*. (Midwife 1, Focus group 1)

#### Influenced/affected by own feelings

The meeting with a suspected or identified woman exposed to violence caused many emotions among the midwives. Different experiences influenced their way of dealing with their own feelings. They could feel lonely in the situation and any action demanded courage. The perpetrator could give a sense of insecurity. The midwives gave more of themselves in the care of a woman who was suspected of being exposed to IPV. It was also perceived as difficult to drop the thoughts about the woman’s situation when work was finished for the day.

*‘It´s patients you met quite a while ago. You don´t forget them. You remember the situation as if it was yesterday. When there has been something. It´s not something you just let go when it´s all over. No, you carry it with you the whole time. And sometimes wonder what happened to them?’*. (Midwife 15, Focus group 4)

## DISCUSSION

The main finding was that, for the in-hospital care midwives considered, it was the responsibility of the midwives working at the ANC to identify violence-exposed women. They renounced their own responsibility and transferred it to their colleagues at ANC. This is in conflict to the Mezey et al.^31^ findings where the midwives felt that all healthcare professionals should have knowledge of the subject and share the responsibility to detect violence. Also, Mauri et al.^30^ showed that midwives, working in both ANC and in-hospital prenatal wards, felt that to ask on routine was necessary. The National Board of Health and Welfare in Sweden recommended already in 2014, that all healthcare professionals in case of suspicion of DV should ask respectfully and privately about the cause of signs or symptoms of violence^28^. This shows clearly that there is a need for support to the midwives working at in-hospital wards, in the form of education and time, to integrate this difficult task into their work.

The midwives working at in-hospital prenatal care might suspect IPV if the pregnant woman was absent from scheduled visits or frequently sought care without obvious medical cause. Previous studies show that diffuse problems such as obscure stomach problems, back problems, anxiety, depression and stress could be interpreted as signs of violence^32,40^. Women living in a dysfunctional relationship with violence were more likely to be higher users of healthcare services^38^, although the response from healthcare in relation to DV is often inadequate and women exposed to violence often remain undetected, pregnant women being no exception^39^. Visible injuries such as bruises were identified as clear signs by the in-patient care midwives, which is also supported by previous research where midwives working at ANCs are interviewed^32^. The respondents showed awareness about that pregnancy-related complications such as hyperemesis, bleeding, or decreased foetal movements, which could occur because of IPV. They also expressed that it is important that all midwives should have up-to-date knowledge of the symptoms and signs of violence, which is supported by the Swedish National Board of Health and Welfare^28^. In contrast, according to earlier research, DV is a social problem that is not included in the midwife’s medical area of expertise^31^. The in-hospital care midwives said that they strived to supporting pregnant women who already were identified as exposed to violence or if there was a suspicion about the pregnant woman having a dysfunctional relationship where IPV occurred. The main hindrances for giving support were both the work-place design and the routines that constituted a barrier. Also, the midwives own emotional state could affect their handling of the situation with the violence-exposed woman. This result shows clearly that the care provider needs to support and take a greater responsibility for the midwives working situation regarding this delicate issue. Knowledge and education about violence against women need to be increased, as supported by the National Board of Health and Welfare in Sweden^28^. Also, there is a responsibility to develop guidelines to follow rests on the healthcare providers. Resources are needed for this gender-based matter.

The midwives working at in-hospital wards expressed that suspicion of DV could be an emotion that arose from the behaviour of the pregnant woman, the partner, or the couple. Earlier research supports this result, i.e. when the partners behaviour is manipulative or controlling^31,32^ and if the woman was quiet, restrained, or suppressed^40^. More education and training are obvious needs to strengthen midwives to handle those situations and have been shown to increase the knowledge and the readiness to ask^41^. However, the detection of violence and referral of victims of violence is low^41^. There is no direct evidence that by asking on routine harms women, and the Cochrane systematic review by O’Doherty et al.^33^ shows that asking on routine increases the identification of women experiencing IPV. However, it is known that when woman leave an abusive relationship it is the time of greatest risk for serious escalation in violence, including homicide. Encouraging disclosure without a functioning system of care in place to help women and children can be harmful.

The participants identified women of foreign origin as a particularly vulnerable group. Language impairment made it difficult to identify violence in close relationships, which is supported by previous research^42,43^. This is a vulnerable group because of the language barrier and limited knowledge about the new country’s laws and judicial system, as well as what the society can offer for support.

To establish a trusting relationship with the pregnant women was, in the present study, expressed as necessary before asking a question about possible violence. This result has been demonstrated in several studies^30,42-44^. Otherwise, the issue can be perceived negatively and cause damage to the midwife’s relationship with the woman^32,42,43^. According to Spangaro et al.^16^, confidence in the midwife was the main reason why the violence-exposed pregnant woman chose to disclose her vulnerable situation. By conveying an openness, the midwife can also work towards gaining the woman’s confidence so that she dares to report any vulnerability. According to Halldórsdóttir and Karlsdóttir^17^, the midwife’s ability to strengthen the woman is part of the interpersonal skills. However, the midwife must have respect for the woman’s self-determination and informed decision^32^. The maternity midwife can, through a respectful approach, inform the violence-exposed pregnant woman about the help that society has to offer and provide telephone numbers, for example the Women’s Support Line’s national support telephone number, for any future help contact. When a midwife suspects that a pregnant woman is subjected to IPV, she is confronted with a very complex and difficult situation. More education and support to in-hospital midwives are needed to strengthen their ability to support women in situations where they have a suspicion of IPV. Routines for how to ask and what to do must be clear to everyone.

Lack of continuity of care between ANC and in-hospital prenatal ward hindered the ability of midwives to engage with the pregnant woman. Midwives working in a hospital have the same responsibility to be aware of violence and to establish a trusting relationship with the pregnant woman^28^. It can be easy for in-hospital midwives to neglect their responsibility because of insecurity about how to handle the delicate situation. Continuity of care must be developed between ANC and in-hospital maternity care. A woman-centred approach must be the key and can be achieved by providing care within a midwifery continuity of care model^45^.

The findings showed that when the midwives had identified a violence-exposed pregnant women, they felt an obligation to offer professional help. They also felt inadequate in their own ability to deal with this sensitive issue. To be able to refer to another professional such as a psychologist, welfare officer, social secretary, or to a woman’s shelter, could give a sense of security. According to Mauri et al.^30^, midwives emphasized the importance of interdisciplinary care and referral to professional help, as they did not consider themselves able to take full responsibility. This highlights the very important issue that the employer has indeed a responsibility to ensure that also midwives working in hospitals should know what kind of professional help is available in these situations.

The in-hospital midwives felt a responsibility to protect the coming child when a pregnant woman was exposed to IPV. As in previous research^31,32,42^, the midwives revealed frustration when the pregnant woman refused help. Like the findings in the study by Finnbogadóttir and Dykes^32^, it emerged that midwives experienced a dilemma between the professional obligation to protect the mother and the coming child, and the woman’s unwillingness to change her situation, as well as the fact that the unborn child was not yet considered a legal person. When there was a child within the family, a notification according to the Social Services Act (SFS 2001: 453)^46^ could be felt as a safety measure, but an act for the best interests of the child did not necessarily mean an improved situation for the mother.

Lack of time was a significant barrier. High workloads resulted in the prioritization of medical measures, which is reinforced by previous research^30,42,43^. Although, the in-hospital midwives tried to handle the situation by prioritizing their tasks. Support from colleagues was felt to be important in dealing with this delicate situation. This is also strengthened by previous research^32^. For midwives working in hospital, the present study highlighted another barrier. This was a lack of a place where they could talk privately with the woman. This was mentioned as a specific hindrance to communicating with women in the hospital setting and may therefore be unique to in-hospital perinatal wards. The organization of hospital care must be easily able to bridge this hindrance if there is a willingness from the organization to solve the problem about privacy.

An additional barrier, which was mentioned by the inhospital midwives, was that education and action plans failed when they detected a pregnant woman exposed to IPV. The midwives felt saddened about the situation, but also a responsibility to act. Furthermore, the midwives felt a lack of feedback in cases where they had acted. They demanded training programs about DV which they mentioned as a way to increase their knowledge and confidence in dealing with IPV. This problem was also noticed by Baird et al.^42^. An easily accessible and clear plan of action should be provided to support the midwives when meeting with a pregnant woman who is exposed to IPV. The action plan should include proposals for possible action and contain contact information for authorities that may need to be involved in supporting the woman.

The participants expressed that they felt alone in dealing with the situation and that action requires courage from the midwife, which is also evident in previous research^32^. Mezey et al.^[Bibr cit0031]31^ showed that personal experience of violence could contribute to making the midwife feel uncomfortable about asking questions on violence in close relationships. Fear of asking the question could prevent midwives from identifying a violence-exposed pregnant woman^44^.

### Strengths and limitations

According to Kruger and Casey^35^, the optimal size for the focus group when the topic is sensitive is four to five participants. However, due to difficulties with the recruitment there were only two people in one of the groups, but the discussion was fruitful. The credibility of presented findings is confirmed by presented dialogue interactions in the focus groups. One limitation is the transferability of the findings, which are limited to the demographic area in which the study was performed.

## CONCLUSIONS

The results showed that the midwives working at inhospital prenatal wards considered that it was primarily the responsibility of midwives at ANC to ask routinely and disclose violence-exposed pregnant women. The midwives had limited experience in dealing with these women but identified several signs and symptoms that could cause suspicion. They felt uncomfortable in the situation and expressed a need for both education and a plan of action. The healthcare providers need to take on their responsibility and ensure education to all personnel, as well as having a clear plan of action when a pregnant woman is assessed as being violence-exposed. All midwives in the pregnant woman’s care network should have basic knowledge of violence in close relationships and a preparedness to support the vulnerable woman in the best possible way.
